# Daily tadalafil for the chronic phase of stuttering priapism: a case report

**DOI:** 10.1186/s12894-018-0368-x

**Published:** 2018-05-31

**Authors:** Paolo Massenio, Nicola D’Altilia, Francesca Sanguedolce, Giuseppe Carrieri, Luigi Cormio

**Affiliations:** 1 Department of Urology and Renal Transplantation, University of Foggia, Foggia, Italy; 2 Department of Pathology, University of Foggia, Foggia, Italy; 3 Bari-Palese, Italy

**Keywords:** Priapism, PDE-5 i, Winter’s shunt, Erectile dysfunction, Tadalafil

## Abstract

**Background:**

Recurrent (stuttering) ischemic priapism is a challenging clinical condition. Frequent recurrences result in frequent hospital admissions whereas treatment with a shunting procedure often results in erectile dysfunction.

**Case presentation:**

A 22-year-old man with stuttering idiopathic priapism developed erectile dysfunction (IIEF-5 score 12) following a Winter’s shunt; he was given tadalafil, 5 mg/daily, for 6 months. This treatment resulted in progressive restoration of erectile function in the 6 months following the shunt as well as in preventing recurrence of priapic episodes over a 24-month follow-up.

**Conclusions:**

This is the first report in literature of chronic treatment of stuttering priapism with a phosphodiesterase-5 inhibitor being able not only to prevent recurrent priapic episodes but also to restore erectile function following a Winter’s shunt.

## Background

Recurrent priapism, commonly known as stuttering priapism, is an unusual form of low-flow priapism that usually results in cavernous ischemia with consequent damage of erectile function. Sickle cell disease is considered the most common cause or stuttering priapism. Another large number of cases are classified as idiopathic, whereas non-erectogenic drugs or neurological disorders are rarely responsible for such condition.

Primary treatment involves corporal aspiration followed by intracavernous injection of sympathomimetics [1]; in case of failure, a shunting procedure becomes mandatory. The most common shunting procedure remains the Winter’s shunt as it is quick and successful in 50 to 65% of cases [2, 3]. Unfortunately, the Winter’s shunt does not prevent recurrences [4, 5] and, on the other hand, leads to erectile dysfunction (ED) when the procedure is carried out within 24 h of priapism onset [6], therefore well before ischemia has led to definite cavernosal damage [7, 8]. Howewer, satisfactory results have been reported preservation of prepriapism erectile function, after 24 h after onset [9]. Herein we describe treating the chronic phase of stuttering idiopathic priapism with tadalafil, 5 mg daily, in order to preventing recurrences and restoring erectile function following Winter’s shunt.

## Case presentation

A 22-year-old man presented to our emergency clinic with a long-standing (5 h) sustained painful erection. He had no history of previous illnesses, trauma, drug intake or previous similar attacks. He reported having a stable heterosexual relationship and that his sustained erection had started outside a sexual encounter. When he was asked if this was the first episode, he mentioned that he had noticed, approximately over the last 18 months, an increase in number and duration of his spontaneous erections; however, given the absence of significant pain, such events were not considered relevant.

On examination, the penis was fully erected with a soft glans. Intracavernous blood sampling was suggestive for hypoxic, low-flow priapism (Ph 7.06; PCO_2_ 14, arterial ref.: 4.5–6.1 kPa; PO_2_ 2.6, arterial ref.: 10–13.5 kPa). Moreover, penile and perineal duplex ultrasound ruled out an arteriovenous fistula. He therefore underwent intracavernous injection (ICI) of etilefrine 5 mg followed by corporeal irrigation/aspiration and again (ICI) of etilefrine 5 mg followed by corporeal irrigation/aspiration; this procedure, however, did not lead to penile detumescence. Meanwhile, peripheral blood analysis ruled out hematological disorders. He was admitted with the diagnosis of low-flow idiopathic priapism and scheduled immediate Winter’s shunting. The procedure was carried out under spinal anesthesia using a 16-G automatic spring-loaded tru-cut needle and led to complete detumescence.

The following morning, the penis was flaccid but the patient reported having had a spontaneous morning erection. He was discharged home but, 2 days later, he presented to our emergency clinic with a long-lasting (4 h) sustained painful erection outside a sexual encounter. Again, intracavernous blood sampling was suggestive for hypoxic, low-flow priapism (Ph 7.00, PCO_2_ 13, PO_2_ 2.4) and again ICI of etilefrine 5 mg and corporeal irrigation/aspiration done twice did not lead to penile detumescence. Therefore, he was admitted and scheduled for multiple (two for each corpus cavernosum) Winter’s shunts resulting in complete detumescence. The urethral catheter, left in place at the end of the procedure, was removed on second postoperative day. Persisting complete penile detumescence and absence of spontaneous erections, the patient was discharged on fourth post-operative day with the advice of avoiding sexual encounter.

At one-week follow-up, the penis was fully detumescent but the patient reported a few episodes of spontaneous tumescence. Therefore, he was allowed to start sexual encounters. At one-month follow-up, he reported several episodes of spontaneous tumescence never reaching rigidity and of prolonged (> 3 h) good tumescence but no penile rigidity during sexual encounters, scoring 12 on International Index of Erectile Function [IIEF-5] questionnaire. Following extensive discussion and a written informed consent, he was given tadalafil, 5 mg daily. At 3-month follow-up, he reported progressive improvement of his erections, both spontaneous and sexually-induced, with an IIEF-5 score of 18. At 6-month follow-up, the IIEF-5 score had reached 22; daily tadalafil was stopped and substituted with tadalafil 10 mg on-demand. At 9-months follow-up, the patient reported having resumed normal spontaneous and sexually-induced erections, with no episodes of prolonged erection nor of tadalafil use; did IIEF-5 score remain at 22. Therefore, he was stopped any treatment. To date, at 24-month follow-up he has normal spontaneous and sexually-induced erections without any drugs; IIEF-5 score remains 22.

## Discussion

The pathophysiology of stuttering priapism is unknown. It has been speculated that downregulation of adrenoreceptors in the cavernous smooth musculature or scarring of intracavernous venules may trigger recurrences [10]. On the other hand, recent studies suggest stuttering priapism to be related to a defective PDE5 regulatory function in the penis, resulting from altered nitric oxide and cyclic guanosine monophosphate signaling mechanisms which control erectile function [11–15]. Specifically, altered vascular homeostasis and oxidative stress could cause endothelial damage with reduced production of endothelial NO, which leads, by a negative feedback mechanism, to downregulation of PDE-5 expression. Under these conditions, cyclic guanosine monophosphate builds up in the corpora cavernosa and cannot be degraded due to lack of PDE5 function, thus leading to prolonged corporal smooth muscle relaxation/priapism episodes. Moreover, erections may also be induced by neuronal NOS. Such mechanisms would explain recurrent priapic episodes as well as damage of erectile function in patients with stuttering priapism.

Due to their erectogenic effect, oral PDE5 inhibitors are commonly used as medical treatment for ED; however, scientific evidence has shown they have a paradoxical effect in alleviating stuttering priapism [15–20]. Preclinical studies suggest daily administration of low-dose PDE-5 inhibitors after the acute period of priapism to upregulate PDE-5 gene expression, to stimulate eNOS expression and to reduce the state of dysfunctional NO pathway [21]. By doing so, such treatment could restore the balance between stimulating and inhibiting factors, thus reducing the episodes of priapism [22]. In a small case series, Burnett and colleagues [18] showed that daily PDE5 inhibitor therapy reduced ischemic priapism episodes in men with stuttering priapism, either idiopathic or due to sickle-cell disease, without modifying erectile function. Moreover, there was no adverse event and only one patient did not respond to treatment. The authors pointed out that such treatment, which should be started under conditions of complete penile flaccidity, was most useful for patients with mild or moderate disease. None of treated patients, however, had undergone a Winter’s shunt.

To our knowledge, our is the first report of long-term administration of tadalafil 5 mg/daily for preventing recurrent episodes of stuttering priapism as well as for treating ED which had developed after having managed a non-subsiding priapism recurrence by a Winter’s shunt. As a matter of fact, findings confirmed the ability of such treatment to preventing recurrent priapic episode over the 24 months follow-up period; moreover, it resulted in progressive restoration of erectile function, with IIEF-5 score raising from 12 to 22 in 6 months and remaining stable over the next 18 months.

## Conclusion

In conclusion, idiopathic priapism is a rare clinical condition. Acute treatment consists in corporal aspiration, intracavernous injection of sympathomimetics and, in case of failure, a shunting procedure. The reported case suggests that prevention of new priapic episodes as well as restoration of erectile function following a Winter’s shunt can be achieved by chronic administration of tadalafil, 5 mg/daily. Such observation could set the basis for more extensive evaluation of such treatment in this unusual condition.

## Abbreviations

ED: Erectile dysfunction
IIEF: International Index of Erectile Function
NO: Nitric oxide
PDE-5: Phosphodiesterase type 5 inhibitor

## References

[1] Broderick GA, Harkaway R (1994). Pharmacologic erection: time-dependent changes in the corporal environment. Int J Impot Res.

[2] Grayhack JT, Mccullough W, O’conor VJ, Trippel O (1964). Venous bypass to control priapism. Investig Urol.

[3] Winter CC (1979). Priapism treated by modification of creation of fistulas between glans penis and corpora cavernosa. J Urol.

[4] Sadeghi-Nejad H, Seftel AD (2002). The etiology, diagnosis, and treatment of priapism: review of the American Foundation for Urologic Disease Consensus Panel Report. Curr Urol Rep.

[5] Levey HR (2012). Medical management of ischemic stuttering priapism: a contemporary review of the literature. Asian J Androl.

[6] Hudnall, Pria 2016 (2017). Advance in the understanding of priapism. Transl Androl Urol.

[7] El-Bahnasawy MS, Dawood A, Farouk A (2002). Low- flow priapism: risk factors for erectile dysfunction. BJU Int.

[8] Pryor, Hehir (1982). The management of priapism. Br J Urol.

[9] Pal (2016). Oucome and erectile function following treatment of priapism: an institutional experience. Urol Ann.

[10] Bochinski DJ, Deng DY, Lue TF. The treatment of priapism- when and how? Int J Imp Res. 2003;15(Suppl 5):S56–S90. 10.1038/sj.ijir.3901078 14551583

[11] Broderick GA, Kadioglu A, Bivalacqua TJ, Ghanem H, Nehra A (2010). Priapism: pathogenesis, epidemiology, and management. J Sex Med.

[12] Mantadakis E, Cavender JD, Rogers ZR, Ewalt DH, Buchanan GR (1999). Prevalence of priapism in children and adolescents with sickle cell anemais. J Pediatr Hematol Oncol.

[13] Bivalacqua TJ, Burnett AL, Graham SD, Glen JF (2010). Priapism. Glenn’s urologic surgery.

[14] Bivalacqua TJ, Burnett AL (2006). Priapism: new concepts in pathophysiology and new treatment strategies. Curr Urol Rep.

[15] Champion HC, Bivalacqua TJ, Takimoto E, Kass DA, Burnett AL (2005). Phosphodiesterase-5A dysregulation in penile erectile tissue is a mechanism of priapism. Proc Natl Acad Sci U S A.

[16] Bivalacqua TJ, Musicki B, Hsu LL, Gladwin MT, Burnett AL (2009). Establishment of a transgenic sickle-cell mouse model to study the pathophysiology of priapism. J Sex Med.

[17] Bialecki ES, Bridges KR (2002). Sildenafil relieves priapism in patients with sickle cell disease. Am J Med.

[18] Burnett AL, Bivalacqua TJ, Champion HC, Musicki B (2006). Feasibility of the use of phosphodiesterase type 5 inhibitors in a pharmacologic prevention program for recurrent priapism. J Sex Med.

[19] Bivalacqua TJ, Musicki B, Champion HC, Burnett AL, Carson IIICC, Kirby RS, Goldstein I, Wyllie MG (2009). Phosphodiesterase type 5 inhibitor therapy for priapism. Textbook of erectile dysfunction.

[20] Burnett AL, Bivalacqua TJ (2007). Priapism: current principles and practice. Urol Clin North Am.

[21] Champion HC (2005). Phosphodiesterase-5A dysregulation in penile erectile tissue is a mechanism of priapism. Proc Natl Acad Sci U S A.

[22] Anele UA, Burnett AL (2015). Nitrergic mechanisms for management of recurrent priapism. Sex Med Rev.

